# “I am my own doctor”: A qualitative study of the perspectives and decision-making process of Muslims with diabetes on Ramadan fasting

**DOI:** 10.1371/journal.pone.0263088

**Published:** 2022-03-04

**Authors:** Siham Bouchareb, Rabab Chrifou, Zohra Bourik, Giel Nijpels, Mohamed Hassanein, Marjan J. Westerman, Petra J. M. Elders

**Affiliations:** 1 Department of General Practice, Amsterdam Public Health research institute, Amsterdam UMC, Vrije Universiteit Amsterdam, Amsterdam, Netherlands; 2 Department of Public and Occupational Health, Amsterdam Public Health research institute, Amsterdam UMC, Vrije Universiteit Amsterdam, Amsterdam, Netherlands; 3 Department of Public Health and Primary Care, Unit Health Promotion, Ghent University, Ghent, Belgium; 4 Department of Ethics, Law and Humanities, Amsterdam Public Health research institute, Amsterdam UMC, Vrije Universiteit Amsterdam, Amsterdam, Netherlands; 5 Endocrine department, Dubai Hospital, Dubai, United Arab Emirates; 6 Department of Epidemiology and Data Science, Amsterdam Public Health Research Institute, Amsterdam UMC, Vrije Universiteit Amsterdam, Amsterdam, Netherlands; Universiteit van Amsterdam, NETHERLANDS

## Abstract

**Background:**

Many Muslims with diabetes choose to fast against medical advice during Ramadan, potentially increasing their risk of acute complications. Patients are often reluctant to disclose fasting to their health care providers, and their needs regarding Ramadan are not met in consultations. For healthcare professionals to provide patient-centred care, it is important to gain more insight into patients’ decision-making process. This study therefore aims to explore how Muslims with diabetes decide whether to fast during Ramadan.

**Methods:**

A qualitative study was conducted consisting of 15 focus groups with Muslims with diabetes within a constructivist paradigm. Convenience sampling was used. All focus groups were transcribed verbatim and analyzed using Braun and Clarke’s reflexive thematic analysis.

**Results:**

Four themes were found to be important in the decision on whether to fast: (1) values and beliefs concerning Ramadan, (2) experiences and emotions concerning Ramadan, (3) the perception of illness, and (4) advice from health care professionals, imams and family. Many participants indicated fasting against medical advice and trusting their subjective assessments on whether they could fast. Moreover, three main stages in the decision-making process for eventually refraining from fasting were identified: (1) the stage where positive experiences with fasting dominate, (2) the stage where one encounters challenges but their determination to fast prevails and (3) the stage where one decides to refrain from fasting after experiencing too many physical difficulties with fasting.

**Conclusions:**

Muslims with diabetes experience autonomy in their decisions on Ramadan fasting. The decision to refrain from fasting often resulted from a difficult and dynamic decision-making process and was often made after participants reached their physical limits. These findings highlight the importance of not only shared decision-making to empower patients to make well-informed decisions on Ramadan fasting but also pre-Ramadan diabetes education to help people with diabetes have a safe Ramadan.

## Introduction

During the Islamic month of Ramadan, Muslims abstain from food, drink, oral medications, sexual activity and smoking from dawn to dusk [1]. Ramadan fasting is a religious obligation for healthy Muslim adults [1]. Although the Quran exempts certain individuals from fasting [2], such as those who are ill, many Muslims with diabetes choose to participate in Ramadan fasting [3–9]. Studies confirmed that approximately 43%–80% of people with type 1 diabetes (T1D) [3, 8, 9] and 79%–95% of people with type 2 diabetes (T2D) fasted at least 15 days during Ramadan [3–6]; according to these studies, they fasted an average of 23–27 and 27 days, respectively.

Ramadan fasting is associated with an increased risk of developing acute complications in some people with diabetes, such as hypoglycaemia, hyperglycaemia, ketoacidosis and dehydration [3, 5]. A recent review [10] of epidemiological studies on T2D and Ramadan fasting has shown a 2.6- to 7.5-fold increased risk of hypoglycaemia during Ramadan compared to the month before Ramadan.

Several international Ramadan-related diabetes management recommendations and guidelines have been published [1, 11–13] to reduce the risk of acute complications. These recommendations and guidelines also provide health care professionals (HCPs) with a risk stratification strategy to identify individuals at risk of developing acute complications due to fasting. According to these guidelines, people with diabetes who fall in a high-risk group should be advised against fasting; however, many individuals who are categorized into a high-risk group choose to fast during Ramadan [14–16]. For example, more than 80% of people with T2D who were identified as high-risk according to the American Diabetes Association risk classification fasted during Ramadan [16]. A case-control study [15] which included a high-risk group who were advised not to fast and a lower-risk group who were permitted to fast reported that 76% of the high-risk group fasted against medical advice. Those who fasted against medical advice were almost four times more likely to break their fast due to hypoglycaemia or other acute complications [15].

Furthermore, a survey study conducted in France [17] and one in Turkey [18] reported that around 35% and 66% of people with diabetes, respectively, did not discuss Ramadan fasting with their HCPs before Ramadan. This phenomenon could increase patients’ risk of acute complications due to not receiving pre-Ramadan diabetes education on fasting safely, including information on potential dose adjustments of glucose-lowering medication. Several qualitative studies [19–24] on diabetes and Ramadan have demonstrated inadequate cross-cultural understanding and communication between HCPs and their patients. Patients’ needs are not met in consultations, they feel that their HCPs lack adequate knowledge and understanding of the significance of Ramadan for Muslims and they lack support and advice on fasting safely [21, 23] which could explain why people with diabetes are reluctant to disclose their wish to fast to their HCPs. It is important to gain more insight into how people with diabetes decide on Ramadan fasting and why many choose to fast against medical advice. Several studies, such as a recently published meta-synthesis [25] of 11 qualitative studies explored the experiences and views of Muslims with diabetes on Ramadan fasting, including their motivation for fasting. However, to the best of our knowledge, no previous qualitative studies have explored in depth the decision-making process of Muslims with diabetes regarding the Ramadan fast. In this study, we explored how Muslims with diabetes decide whether to fast during Ramadan. More knowledge on patients’ perspectives could provide relevant insights into patient-centred care when HCPs discuss Ramadan fasting with Muslims with diabetes.

## Methods

### Study design

We conducted an explorative qualitative study with a phenomenological approach using 15 focus groups to explore and understand the perspectives of Muslims with diabetes on Ramadan fasting. A phenomenological approach aims to understand the essence of social phenomena from those who perceived it [26]. Its underlying constructivist paradigm assumes that multiple realities exist, which are socially constructed. We construct knowledge through our lived experiences [27].

The study is part of the Diabetes and Ramadan project, an educational counselling program that aims to improve diabetes care of people with T2D of Moroccan and Turkish descent who observe Ramadan. Therefore, this study is primarily focused on Muslims with T2D. The Medical Ethics Review Committee of VU University Medical Center (Amsterdam UMC) confirmed that the Diabetes and Ramadan project does not fall under the scope of the Medical Research Act Involving Human Subjects; approval from the committee was therefore not required (2018–165).

### Procedure and participants

**Fig 1:**
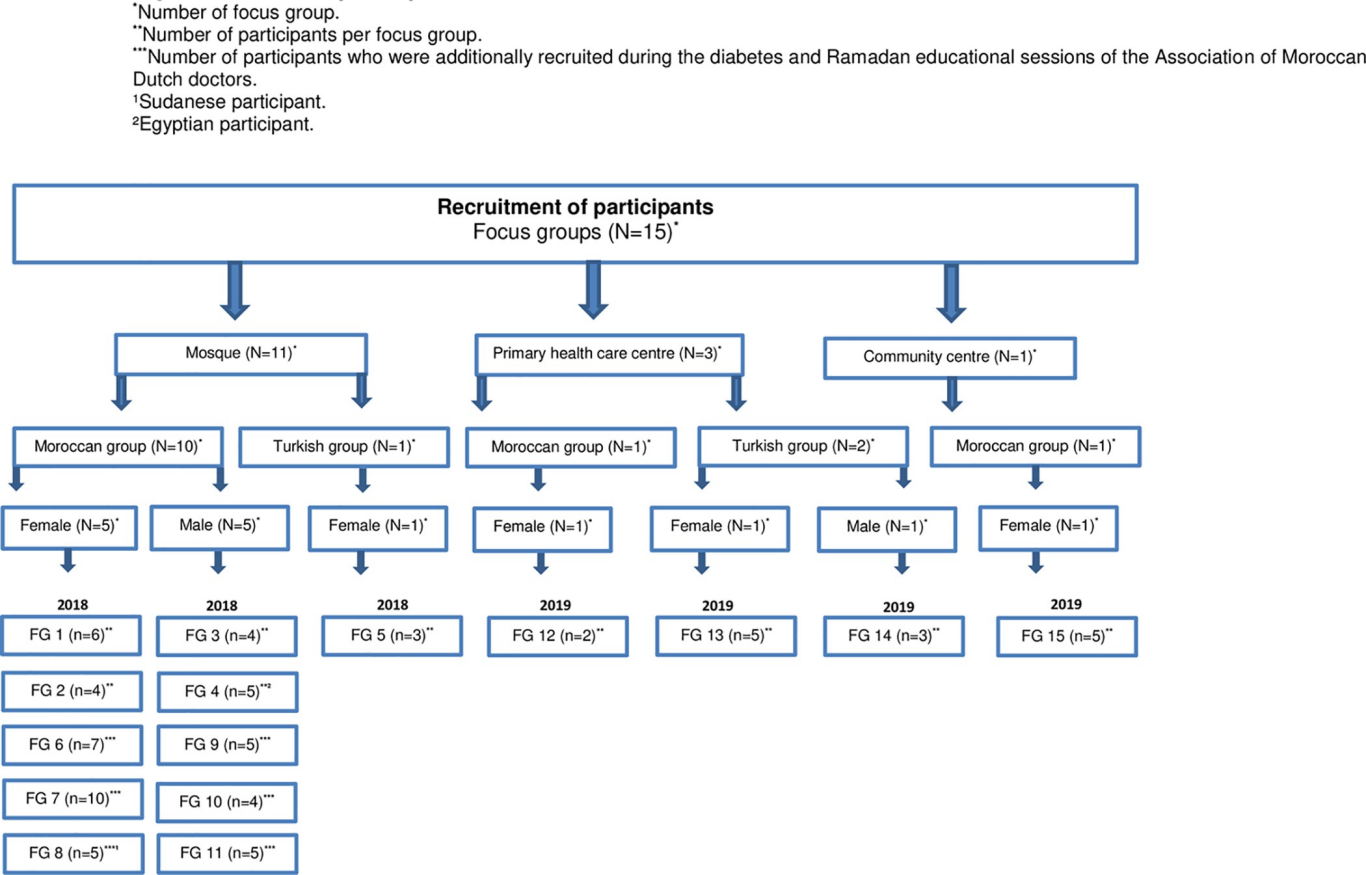
Overview of participant recruitment.

Convenience sampling was used to recruit participants at local mosques, general practices and community centres (Fig 1), primarily during the pre-Ramadan educational sessions, which were organized as part of the Diabetes and Ramadan project. During these sessions, medical students who spoke the participants’ native language helped with the written informed consent. Soon after Ramadan, an appointment for a focus group was scheduled at their local mosque, primary health care centre or community centre. Many participants who provided informed consent could not be reached, mostly due to missing or unreachable phone numbers or their still being on vacation in their native country. Due to the relatively low attendance at the pre-Ramadan educational sessions, we chose to recruit additional participants during the annual diabetes education and screening meetings of the Association of Moroccan Dutch Doctors. We ultimately managed to conduct 11 focus groups in 2018.

In 2019, we chose to conduct additional focus groups to include a more diverse sample, specifically more participants of Turkish descent, as we had conducted only one Turkish focus group in 2018. In addition, to secure input from both groups who would have experienced Ramadan in the same seasonal period, we also conducted two more focus groups with participants of Moroccan descent in 2019.

Participants were initially included if they had T2D and were of Moroccan or Turkish descent. However, a small number of Muslims with T1D, LADA or of other ethnicities (Sudanese and Egyptian) were also interested in participating in the focus groups. Therefore, after discussion within the research team, we decided to expand our inclusion criteria and include those participants. Nevertheless, this study primarily aimed at Muslims with T2D. Table 1 depicts the participants’ characteristics. We included 73 people with diabetes; the vast majority are of Moroccan descent (82.2%) and female (64.4%) and have T2D (93.2%). The mean age is 60.0 years, with a mean diabetes duration of 13.4 years. Many mentioned using oral medication only (49.3%) or in combination with insulin (32.9%). Most participants (72.6%) fasted during Ramadan.

**Table 1:** Characteristics of participants.

		N = 73
Sex, N (%)	Female	47 (64.4%)
Age, mean (SD) in years N (%)		60.0 (9.3)
	35–40 years	2 (2.7%)
	41–50 years	7 (9.6%)
	51–60 years	29 (39.7%)
	61–70 years	22 (31.1%)
	71–80 years	9 (12.3%)
	>80 years	1 (1.4%)
	Unknown	3 (4.1%)
Ethnicity N (%)	Moroccan	60 (82.2%)
	Turkish	11 (15.1%)
	Egyptian	1 (1.4%)
	Sudanese	1 (1.4%)
Diabetes duration, mean (SD) in years N (%)		13.4 (9.2)
	<5 years	13 (17.8%)
	5–10 years	18 (24.7%)
	11–15 years	12 (16.4%)
	16–20 years	13 (17.8%)
	>20 years	13 (17.8%)
	Unknown	4 (5.5%)
Glucose-lowering medication N (%)	Oral medication only	36 (49.3%)
	Insulin only	9 (12.3%)
	Combined oral- and insulin therapy	24 (32.9%)
	No medication	3 (4.1%)
	Unknown	1 (1.4%)
Type of diabetes N (%)	T2D	68 (93.2%)
	T1D	1 (1.4%)
	LADA	1 (1.4%)
	Unknown	3 (4.1%)
Participating in Ramadan fasting N (%)	Yes	53 (72.6%)
	No	20 (27.4%)

### Data collection

The focus groups took place in two large cities (Amsterdam and Den Haag) and a medium-sized city (Leiden) in the Netherlands that all have a relatively large population of individuals with a migration background from May to October 2018 and June to July 2019. We developed a topic guide (S1 Table) inspired by the literature on diabetes and Ramadan [11, 17, 19] and based on the expertise of the research team. All focus were conducted in Moroccan-Arabic, Berber (Tamazight) or Turkish. Two researchers (SB and ZB), who both speak Moroccan-Arabic (SB also understands Berber), conducted the Moroccan focus groups: ZB, who is experienced in conducting focus groups, moderated half of the Moroccan focus groups, while SB, who had received qualitative research training, moderated the other half of the focus groups. In addition, an experienced moderator conducted one of the Turkish focus groups, and a junior doctor conducted two of the Turkish focus groups; both moderators are bilingual in Turkish and Dutch. One researcher (SB) was an interviewer or observer at all 15 focus groups and asked in-depth questions as needed. Before the focus groups began, participants received a brief introduction on the aim and process of the meeting and were asked for permission to audio record the session. The focus groups started with a brief round in which each participant was asked for their name, age, diabetes duration, medication use and whether they fast during Ramadan. The following main topics were explored: the participants’ experiences with Ramadan and fasting, their beliefs on Ramadan fasting and religious exemption, consulting HCPs before Ramadan and pre-Ramadan education. The focus groups lasted 92 minutes on average (range: 43–158 minutes) and were audio recorded and transcribed verbatim directly into Dutch. A transcription agency transcribed around half of the focus groups; the researchers (SB and ZB) and students, all fluent in Moroccan-Arabic and Berber (Tamazight) or Turkish and Dutch, transcribed the remaining half. One researcher (SB) then ensured that the Moroccan-Arabic and Berber transcripts were translated accurately.

### Data analysis

All transcripts were analyzed according to Braun and Clarke’s six phases of reflexive thematic analysis [28–30]. In the first phase, two researchers (RC and SB) familiarized themselves with the data by listening to the audio recorded focus groups, actively reading and re-reading the transcripts. During the second phase, RC and SB independently coded two transcripts using open coding; RC, SB and ZB then discussed these transcripts in detail in order to explore interpretations of the data and to sense-check ideas [31], after which consensus on the codes was reached. All codes derived from these two independently coded transcripts were clustered into an initial coding tree, in which all codes were sorted into categories. Consensus among the research team (MW, PE, RC, SB and ZB) on the initial coding tree was reached. The initial coding tree was then used to code the remaining 13 transcripts. During this process RC and SB remained open to finding new codes. In the third phase, researchers PE and SB clustered the codes into subthemes using a thematic map, and was then discussed back and forth within the research team. The discussions between the researchers were primarily aimed at achieving richer interpretations of meaning [31]. In the following phases, all the data was re-read; codes, subthemes and themes were refined until consensus among the research team on the final subthemes, themes and quotations regarding the decision on whether to fast was reached. Constant comparison was used to search for differences and similarities between participants in their decision on whether to fast. Data was managed and analyzed using MAXQDA 2018. Our study is reported in accordance to the Standards for Reporting Qualitative Research checklist [32].

## Results

### The atmosphere and context of the focus groups

To conduct focus groups in participants’ native language and facilitate the free sharing of experiences and perspectives, we chose homogenous groups in terms of ethnicity and sex [33, 34]. The atmosphere of the focus groups was pleasant; the participants were provided a cup of tea or coffee. Many participants knew one another from the mosque or neighbourhood. Participants engaged with one another and translated the interviewer’s questions into Berber for those who were not fluent in Moroccan-Arabic. They seemed genuinely interested in one another’s experiences, offered one another advice and could, in many cases, disagree with one another. The participants often seemed free to tell jokes, and much of laughter emerged during these sessions. Sometimes the atmosphere felt as if a group of friends were gathered to catch up with one another.

### Reflexive thematic analysis

During the focus groups, it became clear that deciding to refrain from fasting was often difficult for many of the participants. We identified factors related to the decision on whether to fast. These factors are described as four themes: (1) values and beliefs concerning Ramadan, (2) experiences and emotions concerning Ramadan, (3) the perception of illness and (4) advice from HCPs, imams and family. These four themes are clarified in the following section and illustrated with quotations from the respondents, who are identified by their age, sex, ethnicity and whether they fasted during Ramadan for context.

### The decision-making process

**Fig 2:**
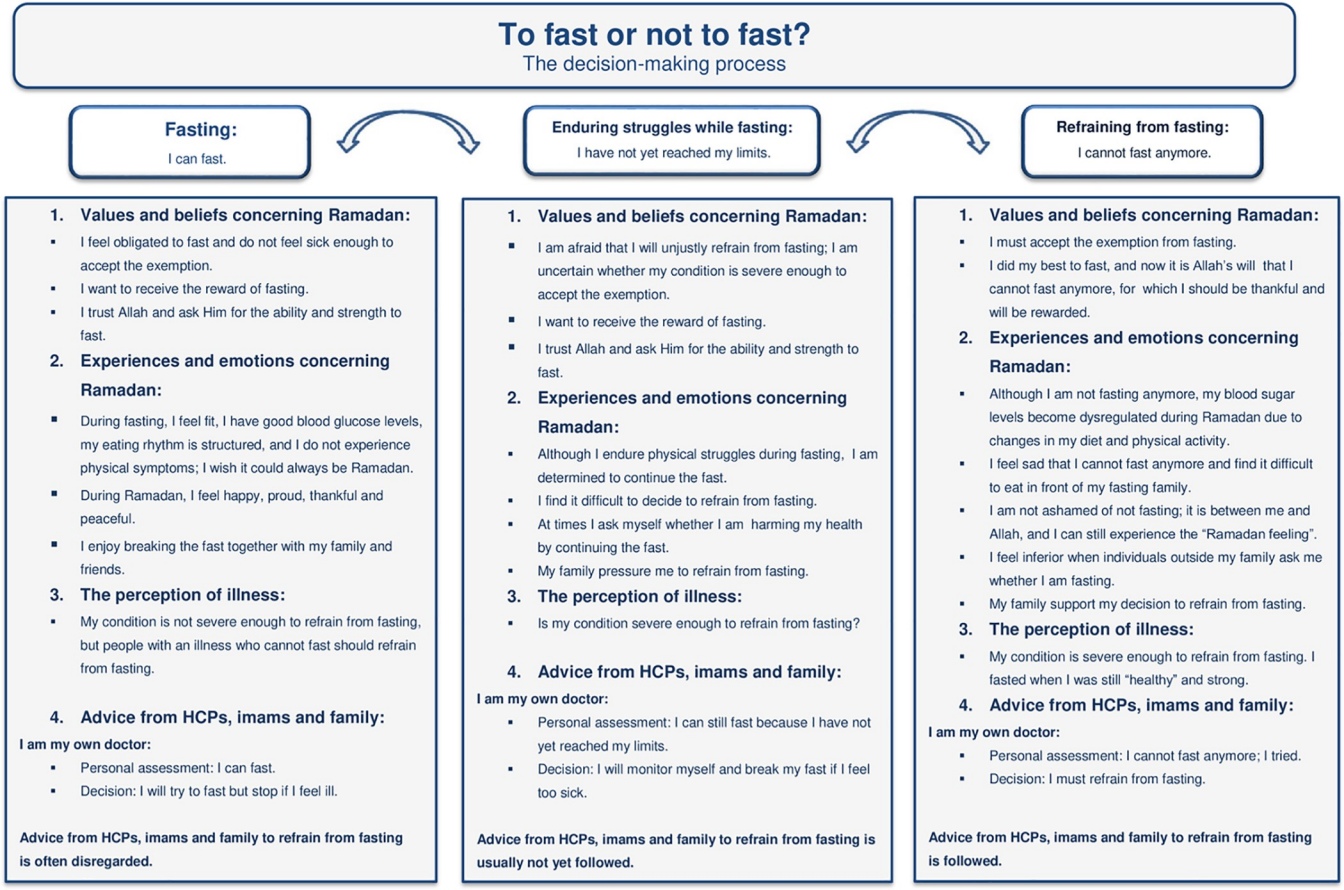
The three stages in the decision-making process for whether to fast according to the four identified themes.

We classified three main stages in the decision-making process for eventually refraining from fasting (Fig 2): (1) the stage where participants have predominantly positive experiences with fasting, feel capable of fasting and therefore chose to fast; (2) the stage where participants endure challenges during fasting but feel that they can still fast, determining to try to fast but willing to break their fast if they feel too ill; and (3) the stage where participants cannot fast anymore due to having reached their limits and decide to refrain from fasting. The participants indicated that this decision-making process for eventually refraining from fasting takes time and is often dynamic. For example, some participants refrained from fasting for a year or a few years before trying to fast again; some of these participants had positive experiences with their renewed attempts at fasting and thus continued to fast. Fig 2 demonstrates the three stages individuals undergo, written from the participants’ perspective according to the four main themes described above.

### Values and beliefs concerning Ramadan

Many participants clarified that people with an illness who cannot fast are exempted from fasting, whereas those who would harm their health through fasting would be committing "suicide" (i.e., committing a sin). A few participants were concerned about committing a sin if they unjustly refrained from fasting. They mentioned feeling uncertain about whether their conditions were severe enough to exempt them from fasting. Some participants who believed themselves incapable of fasting reported having accepted the exemption from fasting; it was considered Allah’s will that they could not fast anymore:

“*My doctor tells me every Ramadan not to fast*… *both my general practitioner (GP) as well as my dietician*… *but I want to fast*, *just like every Muslim*… *you want to fulfil your religious obligations*… *but now that fasting is really not possible anymore*… *it is the will of Allah” [59*, *F*, *Moroccan*, *non-fasting]*.

Participants also expressed their trust in Allah for the ability and strength to fast:

“*This is why I say if a person has imaan [faith in Allah] here [points to her heart] and intends to do something*, *Allah will not disappoint them*. *When I perform my dawn prayer*, *I ask Allah*, *‘One thing I ask from you is to help me fast during Ramadan’” [42*, *F*, *Moroccan*, *fasting]*.

Some participants wanted to receive the reward of fasting, while others mentioned that those who refrained from fasting were also rewarded for accepting the exemption.

Participants believed that fasting was beneficial to their health, especially that fasting purifies and heals the body. Their ideas on the health benefits of fasting originate from various sources, including the Quran.

### Experiences and emotions concerning Ramadan

The participants shared their experiences and emotions of physical, mental and social wellbeing regarding Ramadan. Participants in the first stage shared positive experiences with fasting, which were described as the absence of physical symptoms, feeling fit, losing weight, having stable blood glucose levels and performing daily activities. A few participants expressed their wish for Ramadan fasting throughout the year; they felt good during Ramadan, and it allowed them to structure their diet habits and prevent overeating:

“*(*…*) I feel good during Ramadan*. *As far as I am concerned*, *it should be all-year-round Ramadan; that is what I want*. *As soon as Ramadan is over*, *we just keep eating*. *Food also plays an important role*… *we fill the stomach until it explodes [laughs]” [62*, *M*, *Moroccan*, *fasting]*.

Not all participants had positive experiences. Some mentioned feeling tired, weak and dizzy or suffering from objectified hypo- or hyperglycaemia. Some even continued fasting despite developing symptoms of objectified hypo- or hyperglycaemia:

“*I nearly died*. *My children were also very shocked*… *I had symptoms just before iftar; there was one hour left*. *I lay down and did nothing else*. *In this way*, *I managed to remain fasting*… *but I regret it*. *I regret it*! *When I checked my sugar level*, *it was low”*.

*I*: “*What was your sugar level at the time*?*”*

“*It was 4 mmol/l (72 mg/dl)” [39*, *F*, *Moroccan*, *fasting]*.

In addition, participants who did not fast mentioned enduring challenges during Ramadan due to changes in their diets, sleep patterns and physical activity. For instance, some reported eating less during the day since their family was fasting:

“*No*, *I do not fast*. *It is the third or fourth year that I have not fasted*… *but I spend Ramadan as if I am fasting a little bit because my family is fasting*…*our rhythm also gets confused [laughs]”*.

*I*: “*Do you minimize something*?*”*

“*Yes; you reduce somewhat*, *and you sleep less*… *you eat less during the day*… *[therefore] you still get too low sugar levels” [41*, *F*, *Moroccan*, *non-fasting]*.

Although some were worried about the possible health risks of fasting at times, most participants who could fast expressed feeling happy, grateful, proud and calm during Ramadan. They also reported enjoying the "Ramadan feeling" with their family and friends.

In contrast, some participants expressed feeling sad, guilty or ashamed when they were not fasting. Some found it difficult to be seen eating by their fasting relatives, and therefore avoided eating during the day. Some indicated not eating in front of fasting individuals out of respect:

“*It has been two years now that I have not fasted*, *and I find it very difficult when my children watch me eat*, *even though they know that I am exempt*… *nevertheless*, *it is still difficult to have to eat*. *When I am eating*, *I feel*… *sometimes my wife asks me whether I would like tajine; I say no*, *I do not feel like it*. *I only drink water or a cup of tea*, *and the rest of the day*, *I am basically fasting” [82*, *M*, *Moroccan*, *non-fasting]*.

Not everyone felt ashamed. Some indicated that the decision on whether to fast was between themselves and Allah. Nevertheless, participants reported that questions from individuals outside the family concerning whether they were fasting were experienced as unwanted interference, and some felt inferior in such a situation.

*R1*: “*No but look*… *they know you are ill*… *but when they run into you or they call you [high-pitched degrading voice]*, *‘Hey*, *As-salaam alaikum [peace be upon you]*… *are you fasting or not*?*’*… *If I fast*, *am I fasting for you or someone else*? *Good heavens*! *I fast for myself*… *it is between me and Allah*… *they make it worse”*.

*R2*: “*They make you feel more ill*…*”*

*R1*: “*They make you feel*… *that you are inferior*… *do you understand*?… *as if you are no longer a complete person*, *as if you are less” [59*, *F*, *Moroccan*, *non-fasting]*.

### The perception of illness

Participants shared their opinions on conditions that were perceived to be severe enough to refrain from fasting. Feeling healthy or young, using only oral medication and the absence of comorbidity or diabetes-related complications were viewed as conditions under which fasting was permissible:

“*Our diabetes is not in an advanced stage; we can still fast*, *but if you are in an advanced stage or if you have pain*, *you cannot fast” [53*, *F*, *Moroccan*, *fasting]*.

Participants also stated that individuals should not fast if they are physically incapable of fasting, experiencing severe physical symptoms, suffering from hypo- or hyperglycaemia, using insulin or in a life-threatening situation. Those who refrained from fasting added that being diagnosed with cancer and fearing kidney problems or other health problems due to fasting were reasons for not participating in Ramadan fasting:

“*I have been fasting for 14 or 15 years [with diabetes]*, *but I was still healthy then*. *However*, *now that my glucose drops in the afternoon*, *I cannot stand up; it happens in the evening when it is almost iftar–about 2 to 3 hours before iftar–then it drops*, *but I do not faint*. *Now*, *I cannot fast anymore*. *I cannot remain fasting all day long*. *I ask Allah to forgive us” [75*, *M*, *Moroccan*, *non-fasting]*.

One participant on hormone therapy for breast cancer reported struggling during Ramadan fasting but continuing to fast out of fear of Allah. She felt uncertain about whether her condition was severe enough to refrain from fasting and whether she would unjustly refrain from fasting. This participant became emotional when her fellow focus group members expressed support:

*R1*: “*I find it difficult; I get tired–no*, *not tired–I lose a lot of weight*. *After Ramadan*, *I feel that I have no energy anymore” [sounds sad and ill]*.

*R2*: “*You feel empty”*.

*R1*: “*Yes”*.

*R3*: “*Her energy is gone; her body is out of energy”*.

*R4*: “*But why do you fast*? *Do you want to kill yourself*? *Allah gave you*…*”*

*R1*: “*I am afraid–afraid of Allah”*.

*R4*: “*Why are you afraid*?*”*

*R5*: “*Worship does not include only fasting”*.

*R4*: “*Darling*, *Allah knows what you are going through*. *You have grounds*… *but why–why*? *You have your children*… *you should not cry”*.

*R1*: *[emotional] [60*, *F*, *Moroccan*, *fasting]*.

Those who refrained from fasting indicated that they attempted to fast again before eventually deciding to refrain from fasting permanently:

“*No*, *I do not fast; I am not able to fast anymore”*.

*I*: “*How many years have you refrained from fasting*?*”*

“*Since I have [been diagnosed with] diabetes*… *Every Ramadan I try to fast again*… *but I am not able to fast anymore”*.

*I*: “*So*, *as you said earlier*, *it has been 12 years since you last fasted*?*”*

“*Yes*, *but every Ramadan*, *I attempt to fast*… *but I also have other illnesses*, *not only diabetes”*.

*I*: “*Nevertheless*, *you still try to fast*?*”*

“*Yes*, *I always want to try*… *but I do not succeed” [55*, *F*, *Moroccan*, *non-fasting]*.

### Advice from HCPs, imams and family

Many participants mentioned that they discussed fasting with their HCPs before Ramadan and indicated that their HCPs pro-actively initiated the conversation on Ramadan fasting. Those who did not discuss fasting with their HCPs self-adjusted their medication during Ramadan. One participant felt that HCPs should initiate the conversation on Ramadan fasting. Different attitudes towards HCPs’ advice were expressed: Some respected their HCPs’ advice against fasting, even though they often disregarded that advice, whereas others felt that HCPs advised patients with diabetes against fasting too quickly. A few participants preferred advice from an Islamic HCP, while others mentioned that the HCP’s religious or ethnic background was insignificant. A few even mentioned that they would disregard a Muslim HCP’s advice against fasting.

Most participants whose HCPs advised them not to fast reported choosing to fast against medical advice, implying that they were their own doctors and relied on their own judgement on whether they could fast:

“*The doctor tells you to eat–that you cannot fast*. *He tells you to eat [and] that fasting is dangerous for your kidneys and eyes*. *But I say that I am my own doctor*. *I will try it; if I can*, *then I will fast*, *and if not*, *then it is the will of Allah” [69*, *M*, *Moroccan*, *fasting]*.

Most informed their HCPs that they wanted to try to fast and would break their fast if they felt ill. Others indicated that they had not informed their HCPs of their decision to fast against medical advice:

“*Yes*, *indeed*, *I also fast*. *My previous GP was a Dutch physician*, *and he always advised me not to fast*. *I told him I would not fast*, *but I fasted anyway [laughs]”*.

*I*: “*We indeed hear that more often in Turkish patients with a Dutch GP”*.

“*Yes*, *it could be because he does not understand me*. *He has different values*, *norms and traditions” [59*, *F*, *Turkish*, *fasting]*.

Some participants mentioned consulting an imam when their HCPs advised them against fasting. The imam told participants that they were their own doctors (i.e., they could decide whether they could try to fast):

"*The imam said that I am my own doctor and that I could first try to fast*. *If I cannot handle it then it is fine*, *but I at least have tried it that month… From that moment on*, *I have been fasting…" [62*, *M*, *Moroccan*, *fasting]*

Conversely, others were advised in a consultation or lecture to follow their doctors’ advice and not harm their health due to fasting. Two participants mentioned refraining from fasting after the imam disclosed that they would sin if they fasted against medical advice.

“*(…) I requested her (a relative in Morocco) to ask a sheikh (Islamic scholar) in Saudi Arabia for advice… that I would like to fast*, *I do not dare to eat*, *I have never eaten during Ramadan… She (a relative in Morocco) asked me to tell her exactly what the doctor told me*. *I told her that the doctor said*: *‘do not fast*, *the days are long and fasting with diabetes is a “silent killer”‘*. *She told the sheikh this and sent me the audio-recording (of his reply)*. *He said*: *‘you disobey Allah’*, *‘you disobey Allah’*, *‘you disobey Allah’*, *he said it three times*. *The next day*… *my daughter argued with me that I should not fast*… *I was scared when he said ‘you disobey Allah’ [laughs]*. *So I have eaten/ refrained from fasting [the last 9 days of Ramadan]” [62*, *F*, *Moroccan*, *non-fasting]*.

While others did not follow the imams advice to refrain from fasting.

“*He (imam) said*: *‘You people have diabetes*, *you must refrain from fasting*, *Allah has exempted you from fasting*. *We cannot tell him that we have strong faith in Allah*. *We make our own decision*. *If we get tired*, *then we will break our fast” [49*, *F*, *Sudanese*, *fasting]*.

Some participants also reported that their families advised them not to fast and sometimes argued with them about their decision to fast against medical advice.

“*I always have a discussion with my husband about this*. *He does not want me to fast because I get sick*.

*(…) I thought to myself*: ‘*I want to fast*, *do not interfere*.*’*

*If it (fasting) gets too hard*, *I will not fast” [53*, *F*, *Turkish*, *non-fasting after trying to fast the first five days of Ramadan]*.

## Discussion

This qualitative study explores Muslims’ decision-making process for whether to fast during Ramadan. We found that Muslims with diabetes experience a high degree of autonomy in their decisions on Ramadan fasting. Personal values, beliefs, emotions, perceptions of illness and previous experiences with Ramadan fasting were found to influence the decision-making process. Moreover, deciding to refrain from fasting is often difficult, and in many cases, only made after participants have reached their physical limits during fasting. Three main stages in the decision-making process for eventually refraining from fasting were identified: (1) the stage where individuals have positive experiences with fasting and therefore choose to fast, (2) the stage where individuals endure challenges but remain determined to fast and (3) the stage where individuals refrain from fasting after reaching their limits. Our results suggest that the decision-making process where one stage follows another during the course of diabetes does not move in one direction; patients may attempt to start fasting again before deciding to refrain from fasting permanently. We discuss these three stages in more detail below.

Participants in the first stage reported positive effects of fasting on physical and mental well-being, and some perceived fasting as beneficial to their overall health. The reported positive effects are consistent with those of previous qualitative studies on diabetes and Ramadan [19–21, 23, 24, 35]. Fasting during Ramadan was found to have health benefits regarding cardiometabolic risk factors in healthy individuals [12, 36, 37]. However, these physical benefits in people with T2D are still not very evident. A few studies [4, 38, 39] found a modest reduction in HbA1c, LDL-cholesterol and weight during Ramadan which were not sustained long after Ramadan. Studies on people with diabetes have shown that participating in Ramadan fasting can be beneficial to mental well-being, for instance, by reducing anxiety, depression and stress, which could be associated with the spiritual and social benefits of fasting during Ramadan [1]. Conversely, other studies found that Ramadan fasting can negatively affect mental well-being, for example, due to decreased energy levels, increased irritability and fear of complications [1].

Participants in the second stage disclosed enduring physical challenges during fasting, such as symptoms of potential hypoglycaemia or objectified hypo- or hyperglycaemia. Nevertheless, most continued their fast as they wanted to try to fast for as long as possible. This finding is consistent with two observational studies, one in France [17] and one in Saudi Arabia [16], on people with diabetes, which found that about one-third of their participants refused to break their fast despite suffering from hypoglycaemia, especially when their symptoms developed “just” before the time of breaking the fast (*iftar*). How some of our participants coped with their physical symptoms may be dangerous since small signs of discomfort can quickly develop into a hypoglycaemic coma. These efforts to overcome adverse effects without breaking the fast may also imply inadequate knowledge of diabetes self-management, which raises concerns and underlines the importance of pre-Ramadan diabetes education. Previous studies have suggested that Muslims with diabetes have poor knowledge of diabetes self-management during Ramadan [17, 40]. Participants also expressed their need for more information on participating in Ramadan fasting safely [20, 24].

Finally, participants in the third stage decided to refrain from fasting, mostly after reaching their physical limits, such as recurrent hypoglycaemia. The participants’ search for their physical limits before refraining from fasting might be due to their uncertainty about whether their conditions are "serious" enough to exempt them from fasting. The Quran states that people with an illness are exempt from fasting but does not specify which illnesses are exempted [41]. Islamic jurisprudents, in consultation with medical experts, have specified criteria and conditions under which individuals are exempted from fasting [41], including those stated in the International Diabetes Federation and the Diabetes and Ramadan International Alliance guidelines [1]. However, some people with diabetes who do not experience severe physical difficulties do not perceive themselves as having an illness. They may therefore believe that their chronic condition would not inhibit them from fasting [18, 21, 24]. Another explanation might be the social and religious importance of participating in Ramadan fasting. Fulfilling the religious obligation of Ramadan fasting allows Muslims to feel connected to the Islamic community, which is vital for their religio-cultural identity [41]. Our participants also expressed feeling confident and relying on Allah for the strength to fulfil the Ramadan fast, which is consistent with qualitative studies on Muslims with diabetes conducted in Egypt [24], Malaysia [19] and the UK [21].

In contrast to previous studies [18–21, 41], many participants mentioned discussing fasting with their HCPs before Ramadan. Some participants even indicated that their HCPs initiated the conversation on Ramadan. The highly structured diabetes care in the Netherlands might explain this phenomenon. The Dutch Diabetes Federation and the Dutch College of General practitioners started to pay special attention to the subject in the last few years, which might have had an effect. We cannot exclude that we have a selection bias since we recruited about half of our patients from GP practices that participated in the Diabetes and Ramadan project and could therefore be more aware of counselling patients on Ramadan. Although the project was recent, practices that participate in such a project might be more inclined to provide religion- or culture-related care.

Consistent with previous studies, most participants fasted against medical advice [15, 17]; they sensed a high degree of autonomy in making their own decisions and seemed to rely on their subjective assessments on whether they could fast. Participants’ decision to fast seems to be related to the degree of confidence of one’s ability to fast, which may be related to participants’ prior experiences with fasting and their trust in Allah’s help to fulfil Ramadan fasting. Moreover, religious, social and health-related factors seemed to motivate participants regarding the fast. This is in accordance with the theory of planned behavior [42] which, states that the perceived behavioral control, and behavioral intention, can predict behavioral achievement. People’s behavior is strongly influenced by their confidence in their ability of performing the behavior of interest (i.e., by perceived behavioral control) [42].

Interestingly, many participants reported fasting against medical advice without experiencing adverse effects. Several observational studies [43–47] have demonstrated that people with diabetes who have received pre-Ramadan diabetes education can fast safely. Studies [17, 22, 48] have also indicated that HCPs lack specific knowledge and cultural competence to discuss Ramadan fasting with their patients. This lack of cultural competence might discourage patients from discussing Ramadan fasting with their HCPs, which could increase patients’ risk of adverse effects due to fasting without adequate knowledge of diabetes self-management. An open conversation through shared decision-making could avoid fasting without disclosure, or secret fasting, and its potential health risks.

Our findings suggest that HCPs’ and imams’ advice on whether or not to fast sometimes differed. Some participants reported that imams advised them to follow their doctors’ advice, while others were advised to assess whether they could fast themselves. As a few participants indicated, it would help in the decision-making process if advice from HCPs and religious leaders were consistent. Furthermore, participants who did not fast reported adverse emotions, such as sadness. Imams could reflect on this phenomenon and advocate the religious benefits of accepting the exemption, which might be helpful for some individuals who find it difficult to accept not participating in the Ramadan fast. Being aware of these emotions concerning not being able to fast and reflecting on them might help HCPs discuss Ramadan fasting with their patients.

### Strengths and limitations

To the best of our knowledge, this is the first qualitative study that explores the decision-making process of Muslims with diabetes for Ramadan fasting. Here we discuss the trustworthiness of this qualitative study based on its credibility, dependability and transferability. First, we addressed its credibility by ensuring that our participants could express themselves and elaborate on their experiences freely by conducting the focus groups in Moroccan-Arabic, Berber (Tamazight) or Turkish. Although the moderators did their best to ensure that all participants felt free to share their perspectives and experiences, for example, by explicitly stating that there were no wrong answers and the importance of respecting each other’s point of view. We cannot exclude that the more introverted participants have shied away from expressing their true opinions and therefore potentially leading to social-desirability bias. Another strength is that the focus groups were accessible since they were conducted in a familiar environment for the participants, such as a local mosque, and separate focus groups for both sexes and ethnic groups. The homogeneity of the focus groups could have resulted in the rich data as it empowers participants to express themselves freely [33, 34]. It is a limitation that we recruited participants using convenience sampling. Nevertheless, we managed to include a diverse sample regarding age, sex, diabetes duration, fasting and non-fasting participants and the degree of (health) literacy. We maximized the study’s dependability by including two researchers (RC and SB) in the coding process, where the coding of the first two transcripts was performed independently, in order to achieve richer interpretations of meaning. Moreover, three members of the research team (RC, SB and ZB) are Muslim and have a Moroccan background, which increased the chances of gaining a deeper understanding of our participants’ perspectives and experiences. Two members of the research team (SB and PE) are also physicians, which may have increased our understanding of the participants’ experiences within the clinical context. Nevertheless, these strengths could be a limitation as the researchers’ pre-understanding might have led the results in a certain direction [49]. As to the transferability of our findings to other contexts, it could be a limitation that we included mainly individuals of Moroccan descent and fewer individuals of Turkish, Egyptian or Sudanese descent. The results may therefore not be transferable to other Muslim populations. However, a recent meta-synthesis of 11 qualitative studies on the experiences and views of an ethnically diverse Muslim population concerning diabetes and Ramadan fasting supports several of our results [25]. For example, they also identified a group of participants who took a wait-and-see approach, who tried to remain fasting for as long as possible and participants who determined for themselves whether to fast. Another strength regarding the transferability of our findings is that we also included participants who are illiterate or low literate in Dutch, which increases the transferability to a larger population.

### Implications for clinical practice

Muslims with diabetes feel autonomous in their decision to try to fast. This personal decision is primarily based on their beliefs, experiences and perception of illness. Consistent with previous studies [22, 23], we recommend shared decision-making when counselling people with diabetes on whether to fast during Ramadan and collaboration with imams as they could help patients in their decision, act as religious translators and cultural brokers [50, 51], especially to those patients who are not sure whether their medical condition exempts them from fasting. Since the decision to fast is largely based on previous experiences, it might be helpful if HCPs enquired how fasting was after Ramadan and used this information to improve patients’ knowledge and self-management skills. The topic might be broached again before the following Ramadan. The updated International Diabetes Federation and Diabetes and Ramadan International Alliance practical guidelines (1) describe the main components of Ramadan-focused diabetes education. It is worth noting that fasting as a religious practice may even imply a feeling of empowerment and control regarding health [35]. Ramadan could thus present an opportunity for HCPs to empower their patients with diabetes through Ramadan-focused diabetes education to improve their self-management skills [22, 44]. Pre-Ramadan diabetes education is also important for those who have refrained from fasting as their eating and sleeping rhythms change during Ramadan. In addition, the decision to refrain from fasting is not always a definitive one.

## Conclusions

This study builds on previous research on the perspectives and experiences of Muslims with diabetes regarding Ramadan. We found that Ramadan fasting is a personal choice, and Muslims with diabetes experience a high degree of autonomy in their decisions on whether to fast. This personal decision is based on participants’ beliefs, values, emotions, perceptions of illness and previous experiences with fasting. The decision to refrain from fasting is often a difficult one, mostly made after individuals have reached their physical limits. As to recommendations for clinical practice, our study advocates shared decision-making when counselling patients on Ramadan fasting. It is also crucial to educate fasting and non-fasting Muslims with diabetes on self-management during Ramadan.

## Supporting information

S1 Table Topic guide. (TIF)

## Abbreviations

T1D: Type 1 Diabetes
T2D: Type 2 Diabetes
HCPs: Health Care Professionals
GP: General Practitioner

## References

[1] International Diabetes Federation and DAR International Alliance. Diabetes and Ramadan: Practical Guidelines, Brussels, Belgium: International Diabetes Federation, 2021. Available from: www.idf.org/guidelines/diabetes-in-ramadan and www.daralliance.org

[2] The Holy Quran. Surah Al-Baqarah (Chapter 2) verses 183–185.

[3] Salti I, Bénard E, Detournay B, Bianchi-Biscay M, Le Brigand C, Voinet C, et al. A population-based study of diabetes and its characteristics during the fasting month of Ramadan in 13 countries: results of the epidemiology of diabetes and Ramadan 1422/2001 (EPIDIAR) study. Diabetes care. 2004;27(10):2306–11. doi: 10.2337/diacare.27.10.2306 15451892

[4] Babineaux SM, Toaima D, Boye KS, Zagar A, Tahbaz A, Jabbar A, et al. Multi-country retrospective observational study of the management and outcomes of patients with Type 2 diabetes during Ramadan in 2010 (CREED). Diabetic medicine: a journal of the British Diabetic Association. 2015;32(6):819–28. 25581456 10.1111/dme.12685 PMC6681420

[5] Hassanein M, Al Awadi FF, El Hadidy KES, Ali SS, Echtay A, Djaballah K, et al. The characteristics and pattern of care for the type 2 diabetes mellitus population in the MENA region during Ramadan: An international prospective study (DAR-MENA T2DM). Diabetes research and clinical practice. 2019;151:275–84. doi: 10.1016/j.diabres.2019.02.020 30825560

[6] Hassanein M, Hussein Z, Shaltout I, Wan Seman WJ, Tong CV, Mohd Noor N, et al. The DAR 2020 Global survey: Ramadan fasting during COVID 19 pandemic and the impact of older age on fasting among adults with Type 2 diabetes. Diabetes research and clinical practice. 2021;173:108674. doi: 10.1016/j.diabres.2021.108674 33493579 PMC7826018

[7] Malek R, Hannat S, Nechadi A, Mekideche FZ, Kaabeche M. Diabetes and Ramadan: A multicenter study in Algerian population. Diabetes research and clinical practice. 2019;150:322–30. doi: 10.1016/j.diabres.2019.02.008 30779972

[8] Hassanein M, Alamoudi RM, Kallash MA, Aljohani NJ, Alfadhli EM, Tony LE, et al. Ramadan fasting in people with type 1 diabetes during COVID-19 pandemic: The DaR Global survey. Diabetes research and clinical practice. 2021;172:108626. doi: 10.1016/j.diabres.2020.108626 33321160 PMC7836519

[9] Al Awadi FF, Echtay A, Al Arouj M, Sabir Ali S, Shehadeh N, Al Shaikh A, et al. Patterns of Diabetes Care Among People with Type 1 Diabetes During Ramadan: An International Prospective Study (DAR-MENA T1DM). Adv Ther. 2020;37(4):1550–63. doi: 10.1007/s12325-020-01267-4 32144714 PMC7140750

[10] Hassanein M, Hanif W, Malek R, Jabbar A. Changes in fasting patterns during Ramadan, and associated clinical outcomes in adults with type 2 diabetes: A narrative review of epidemiological studies over the last 20 years. Diabetes research and clinical practice. 2021;172:108584. doi: 10.1016/j.diabres.2020.108584 33307133

[11] Beshyah S, Benbarka M, Sherif I. Practical Management of Diabetes during Ramadan Fast. The Libyan journal of medicine. 2007;2(4):185–9. doi: 10.4176/071008 21503243 PMC3078251

[12] Ibrahim M, Davies MJ, Ahmad E, Annabi FA, Eckel RH, Ba-Essa EM, et al. Recommendations for management of diabetes during Ramadan: update 2020, applying the principles of the ADA/EASD consensus. BMJ open diabetes research & care. 2020;8(1). doi: 10.1136/bmjdrc-2020-001248 32366501 PMC7223028

[13] Hassanein M, Al-Arouj M, Hamdy O, Bebakar WMW, Jabbar A, Al-Madani A, et al. Diabetes and Ramadan: Practical guidelines. Diabetes research and clinical practice. 2017;126:303–16. doi: 10.1016/j.diabres.2017.03.003 28347497

[14] Jabbar A, Hassanein M, Beshyah SA, Boye KS, Yu M, Babineaux SM. CREED study: Hypoglycaemia during Ramadan in individuals with Type 2 diabetes mellitus from three continents. Diabetes research and clinical practice. 2017;132:19–26. doi: 10.1016/j.diabres.2017.07.014 28783529

[15] Afandi B, Kaplan W, Al Kuwaiti F, Al Dahmani Kh, Nagelkerke N.J.D. Ramadan Challenges: Fasting Against Medical Advice. J Fasting Health. 2017; 5(3): 133–137.

[16] Ba-Essa EM, Hassanein M, Abdulrhman S, Alkhalifa M, Alsafar Z. Attitude and safety of patients with diabetes observing the Ramadan fast. Diabetes research and clinical practice. 2019;152:177–82. doi: 10.1016/j.diabres.2019.03.031 30946851

[17] Gaborit B, Dutour O, Ronsin O, Atlan C, Darmon P, Gharsalli R, et al. Ramadan fasting with diabetes: an interview study of inpatients’ and general practitioners’ attitudes in the South of France. Diabetes & metabolism. 2011;37(5):395–402. 21478041 10.1016/j.diabet.2010.12.010

[18] Savaş E. Attitudinal Determinants of Turkish Diabetic Patients and Physicians About Ramadan Fasting. Journal of religion and health. 2018;57(1):47–56. doi: 10.1007/s10943-016-0327-3 27830355

[19] Lee JY, Wong CP, Tan CSS, Nasir NH, Lee SWH. Type 2 diabetes patient’s perspective on Ramadan fasting: a qualitative study. BMJ open diabetes research & care. 2017;5(1):e000365. doi: 10.1136/bmjdrc-2016-000365 28761651 PMC5530234

[20] Almansour HA, Chaar B, Saini B. Perspectives and experiences of patients with type 2 diabetes observing the Ramadan fast. Ethnicity & health. 2018;23(4):380–96. doi: 10.1080/13557858.2016.1269156 27998181

[21] Patel NR, Kennedy A, Blickem C, Rogers A, Reeves D, Chew-Graham C. Having diabetes and having to fast: a qualitative study of British Muslims with diabetes. Health expectations: an international journal of public participation in health care and health policy. 2015;18(5):1698–708. doi: 10.1111/hex.12163 24438123 PMC5060874

[22] Amin MEK, Abdelmageed A. Clinicians’ Perspectives on Caring for Muslim Patients Considering Fasting During Ramadan. Journal of religion and health. 2020;59(3):1370–87. doi: 10.1007/s10943-019-00820-y 31104298

[23] Myers PR, Shoqirat N, Allen DH, Dardas LA. Patients with diabetes observing Ramadan: The experience of Muslims in the United States. Diabetes research and clinical practice. 2019;150:282–7. doi: 10.1016/j.diabres.2018.12.011 30633934

[24] El-Rahman MA, Gida NIM, Sobh DE. Experiences and needs of patients with type 2 diabetes during Ramadan: A qualitative study. Journal of Diabetes Nursing. 2019;23.

[25] Liao J, Wang T, Li Z, Xie H, Wang S. Experiences and views of people with diabetes during Ramadan fasting: A qualitative meta-synthesis. PloS one. 2020;15(11):e0242111. doi: 10.1371/journal.pone.0242111 33226993 PMC7682869

[26] Ataro G. Methods, methodological challenges and lesson learned from phenomenological study about OSCE experience: Overview of paradigm-driven qualitative approach in medical education. Ann Med Surg (Lond). 2020;49:19–23. doi: 10.1016/j.amsu.2019.11.013 31871678 PMC6909137

[27] Varpio L, Ajjawi R, Monrouxe LV, O’Brien BC, Rees CE. Shedding the cobra effect: problematising thematic emergence, triangulation, saturation and member checking. Med Educ. 2017;51(1):40–50. doi: 10.1111/medu.13124 27981658

[28] Braun V, Clarke V. Using thematic analysis in psychology. Qualitative Research in Psychology. 2006;3(2):77–101.

[29] Braun V, Clarke V. Reflecting on reflexive thematic analysis. Qualitative Research in Sport, Exercise and Health. 2019;11(4):589–97.

[30] Braun V, Clarke V. One size fits all? What counts as quality practice in (reflexive) thematic analysis? Qualitative Research in Psychology. 2021;18(3):328–52.

[31] Byrne D. A worked example of Braun and Clarke’s approach to reflexive thematic analysis. Quality & Quantity. 2021.

[32] O’Brien BC, Harris IB, Beckman TJ, Reed DA, Cook DA. Standards for reporting qualitative research: a synthesis of recommendations. Academic medicine: journal of the Association of American Medical Colleges. 2014;89(9):1245–51. 24979285 10.1097/ACM.0000000000000388

[33] Stewart DW, shamdasani P.N, and Rook D.W. Focus Groups: Theory and Practice: Sage Publications: Thousand Oak, CA; 2002.

[34] Green J, Thorogood N. QUALITATIVE METHODS FOR HEALTH RESERACH. 3rd ed: SAGE; 2014.

[35] Mygind A, Kristiansen M, Wittrup I, Nørgaard LS. Patient perspectives on type 2 diabetes and medicine use during Ramadan among Pakistanis in Denmark. International journal of clinical pharmacy. 2013;35(2):281–8. doi: 10.1007/s11096-012-9716-1 23354808

[36] Kamal S, Ahmed QS, Sayedda K, Haque M. Effect of Islamic Fasting on Lipid Profile, Total Protein and Albumin on Healthy Muslim Male Subjects of Shri Ram Murti Smarak Institute of Medical Sciences, Bareilly, Uttar Pradesh. The Journal of medical research. 2012;2:407–10.

[37] Shehab A, Abdulle A, El Issa A, Al Suwaidi J, Nagelkerke N. Favorable changes in lipid profile: the effects of fasting after Ramadan. PloS one. 2012;7(10):e47615. doi: 10.1371/journal.pone.0047615 23112824 PMC3480413

[38] Hassanein M, Abdelgadir E, Obaid HA, Ahmed FS, Alsharhan M, Thabit S, et al. Biometric and metabolic changes in patients with diabetes prior, during and after the holy month of Ramadan (ABCD Study). Diabetes research and clinical practice. 2021;173:108678. doi: 10.1016/j.diabres.2021.108678 33516783

[39] Siaw MY, Chew DE, Toh MP, Seah DE, Chua R, Tan J, et al. Metabolic parameters in type 2 diabetic patients with varying degrees of glycemic control during Ramadan: An observational study. J Diabetes Investig. 2016;7(1):70–5. doi: 10.1111/jdi.12374 26816603 PMC4718105

[40] Ahmedani MY, Siddique M. Assessing the awareness and care of people with diabetes related to Ramadan fasting; a-cross sectional study from Pakistan. Journal of diabetes and metabolic disorders. 2020;19(1):29–36. doi: 10.1007/s40200-019-00471-6 32550153 PMC7270441

[41] Ilkilic I, Ertin H. Ethical conflicts in the treatment of fasting Muslim patients with diabetes during Ramadan. Medicine, health care, and philosophy. 2017;20(4):561–70. doi: 10.1007/s11019-017-9777-y 28444477

[42] Ajzen I. The theory of planned behavior. Organizational Behavior and Human Decision Processes. 1991;50(2):179–211.

[43] Ahmedani MY, Haque MS, Basit A, Fawwad A, Alvi SF. Ramadan Prospective Diabetes Study: the role of drug dosage and timing alteration, active glucose monitoring and patient education. Diabetic medicine: a journal of the British Diabetic Association. 2012;29(6):709–15. doi: 10.1111/j.1464-5491.2011.03563.x 22587405

[44] Bravis V, Hui E, Salih S, Mehar S, Hassanein M, Devendra D. Ramadan Education and Awareness in Diabetes (READ) programme for Muslims with Type 2 diabetes who fast during Ramadan. Diabetic medicine: a journal of the British Diabetic Association. 2010;27(3):327–31. doi: 10.1111/j.1464-5491.2010.02948.x 20536496

[45] El Toony LF, Hamad DA, Omar OM. Outcome of focused pre-Ramadan education on metabolic and glycaemic parameters in patients with type 2 diabetes mellitus. Diabetes & metabolic syndrome. 2018;12(5):761–7. doi: 10.1016/j.dsx.2018.04.036 29729978

[46] McEwen LN, Ibrahim M, Ali NM, Assaad-Khalil SH, Tantawi HR, Nasr G, et al. Impact of an individualized type 2 diabetes education program on clinical outcomes during Ramadan. BMJ open diabetes research & care. 2015;3(1):e000111. doi: 10.1136/bmjdrc-2015-000111 26113984 PMC4477147

[47] Tourkmani AM, Hassali MA, Alharbi TJ, Alkhashan HI, Alobikan AH, Bakhiet AH, et al. Impact of Ramadan focused education program on hypoglycemic risk and metabolic control for patients with type 2 diabetes. Patient preference and adherence. 2016;10:1709–17. doi: 10.2147/PPA.S113324 27660420 PMC5019439

[48] Ali M, Adams A, Hossain MA, Sutin D, Han BH. Primary Care Providers’ Knowledge and Practices of Diabetes Management During Ramadan. Journal of primary care & community health. 2016;7(1):33–7. doi: 10.1177/2150131915601359 26294052 PMC5932667

[49] Graneheim UH, Lindgren BM, Lundman B. Methodological challenges in qualitative content analysis: A discussion paper. Nurse Educ Today. 2017;56:29–34. doi: 10.1016/j.nedt.2017.06.002 28651100

[50] Padela AI, Killawi A, Heisler M, Demonner S, Fetters MD. The role of imams in American Muslim health: perspectives of Muslim community leaders in Southeast Michigan. Journal of religion and health. 2011;50(2):359. doi: 10.1007/s10943-010-9428-6 21088896

[51] Padela AI, Gunter K, Killawi A. Meeting the healthcare needs of American Muslims: Challenges and strategies for healthcare settings. Institute for Social Policy and Understanding. 2011/6.

