# Correction: “I am my own doctor”: A qualitative study of the perspectives and decision-making process of Muslims with diabetes on Ramadan fasting

**DOI:** 10.1371/journal.pone.0315525

**Published:** 2024-12-05

**Authors:** The PLOS One Staff

The S1 Table is incomplete. It should have been four pages. Please see the correct S1 Table below.

The publisher apologizes for the error.

## Supporting information

S1 Table (ZIP)
