# Supplementary figures and images for: “I am my own doctor”: A qualitative study of the perspectives and decision-making process of Muslims with diabetes on Ramadan fasting
Source: PLoS One. 2022 Mar 4;17(3):e0263088. doi: 10.1371/journal.pone.0263088 (PMC8896728; doi:10.1371/journal.pone.0263088)

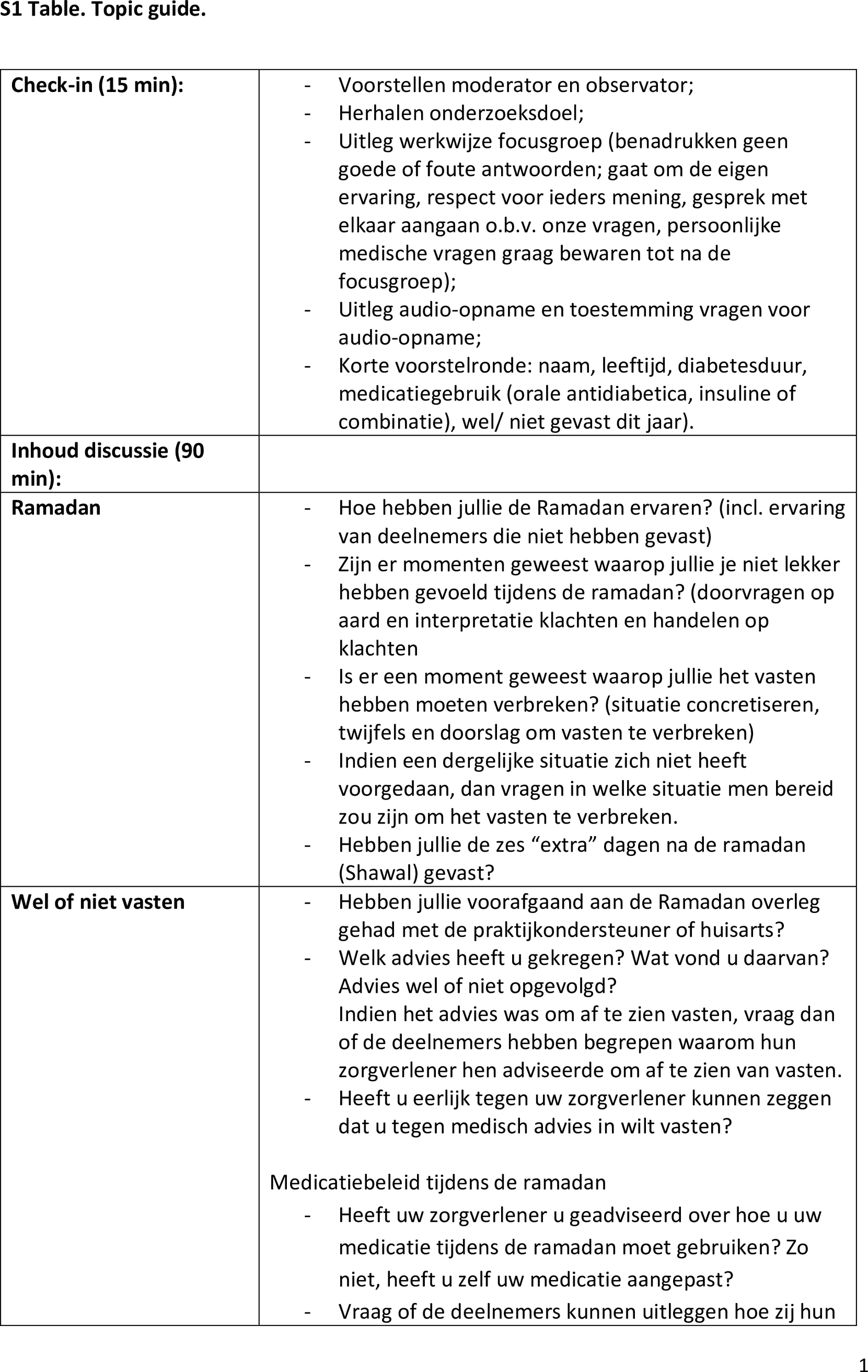

Supplement: S1 Table — (TIF) [file pone.0263088.s001.tif]
