# Supplementary figures and images for: Correction: “I am my own doctor”: A qualitative study of the perspectives and decision-making process of Muslims with diabetes on Ramadan fasting
Source: PLoS One. 2024 Dec 5;19(12):e0315525. doi: 10.1371/journal.pone.0315525 (PMC11620622; doi:10.1371/journal.pone.0315525)

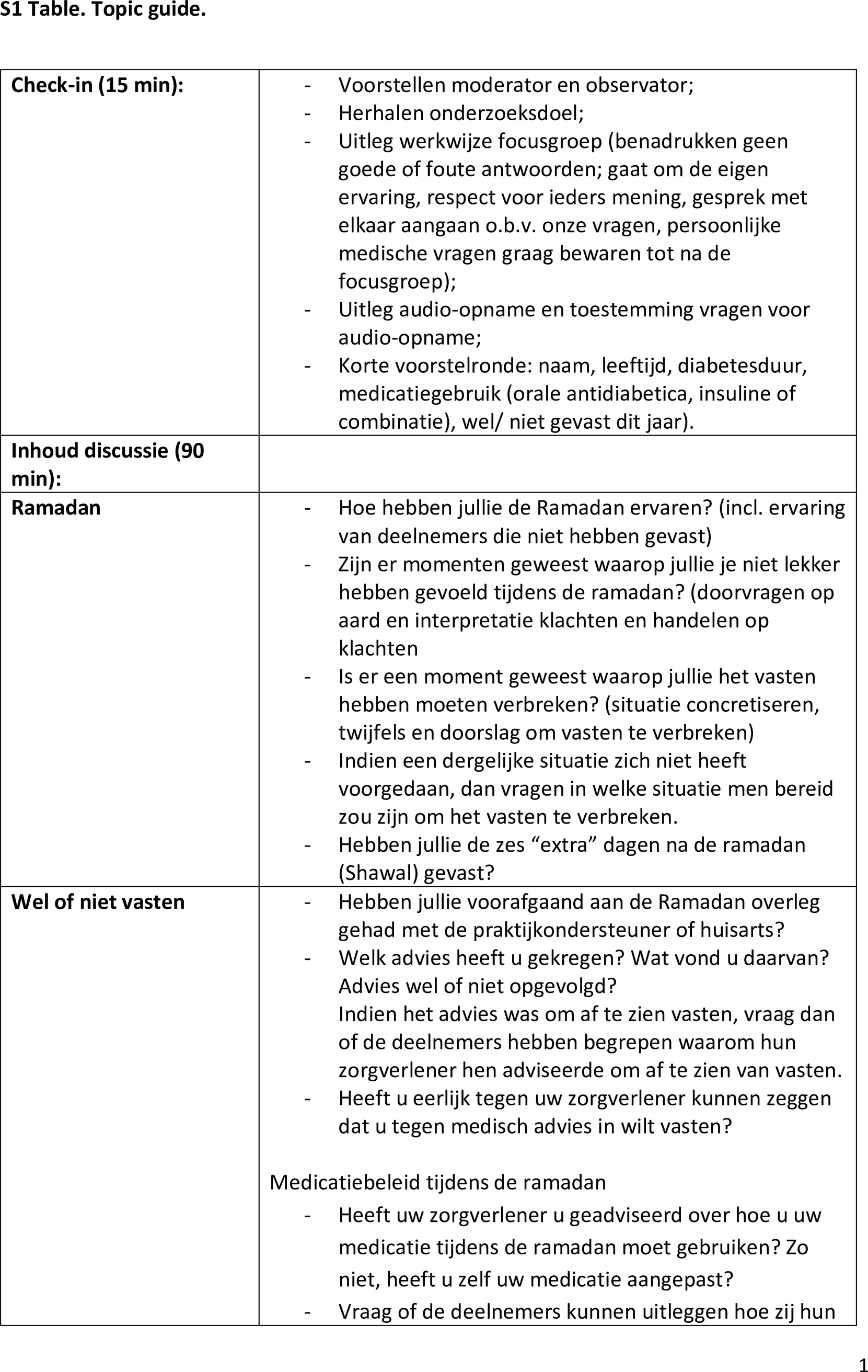

Supplement: S1 Table — (ZIP) [file pone.0315525.s001.zip › S1_Table (1).tif]

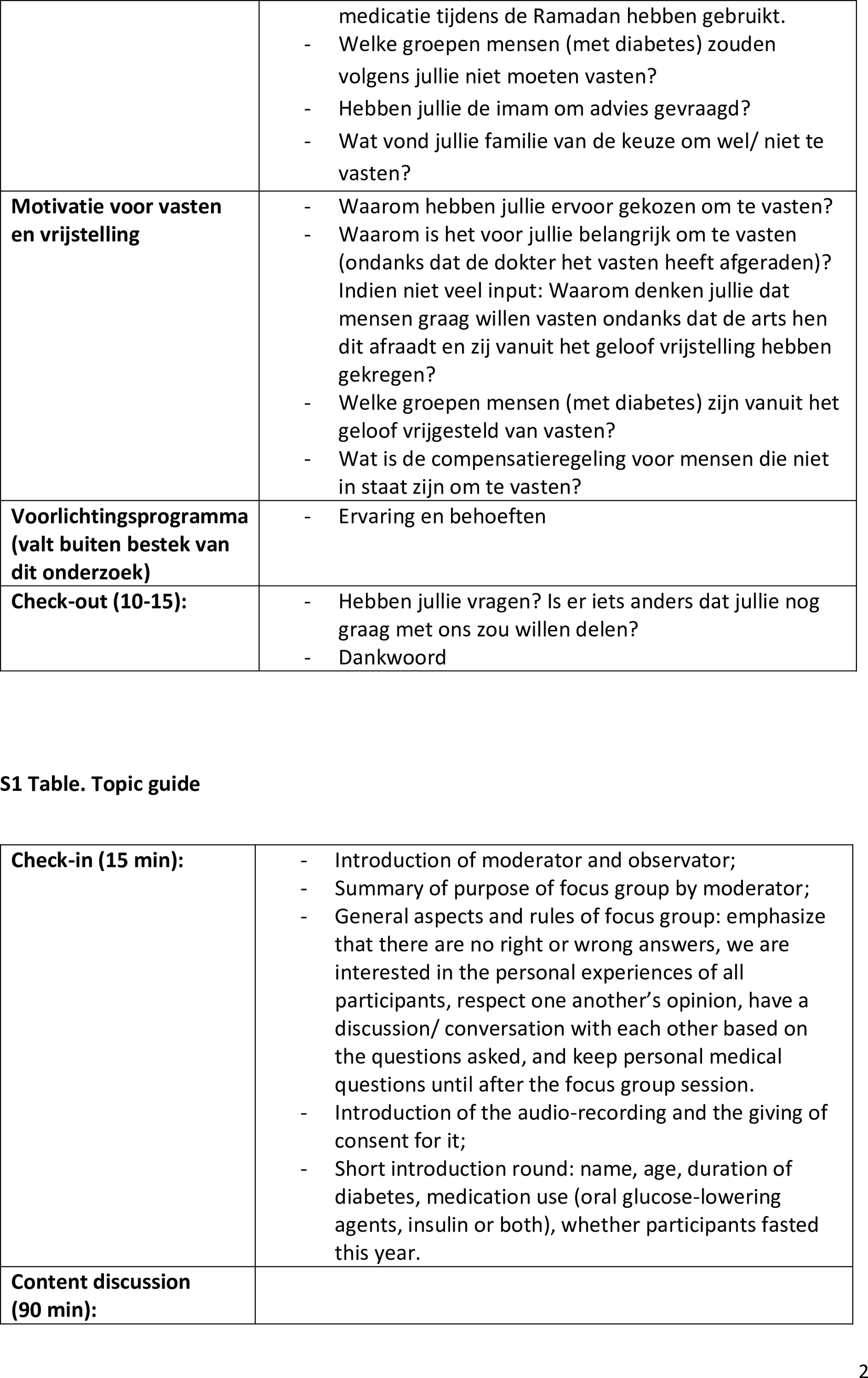

Supplement: S1 Table — (ZIP) [file pone.0315525.s001.zip › S1_Table (2).tif]

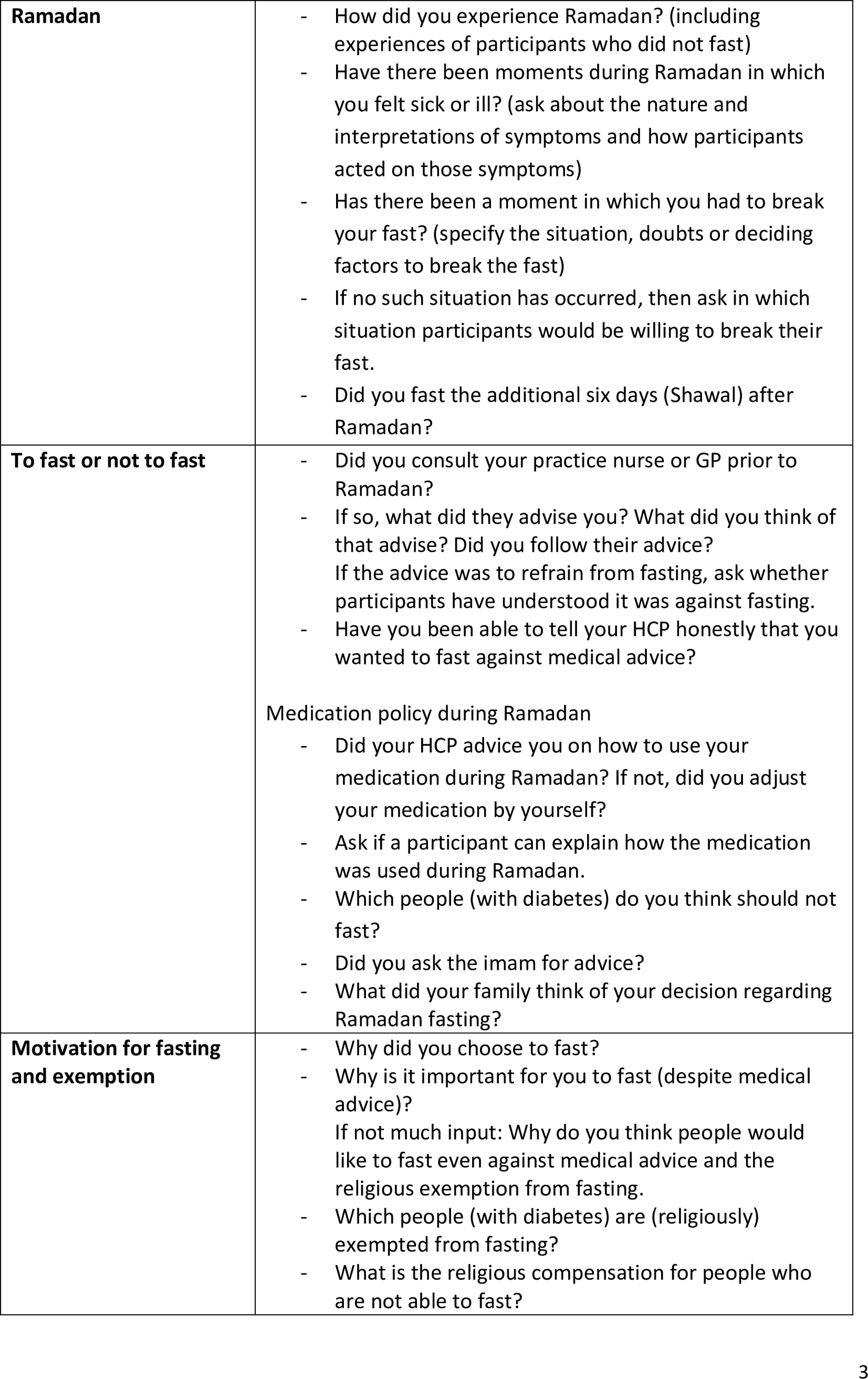

Supplement: S1 Table — (ZIP) [file pone.0315525.s001.zip › S1_Table (3).tif]

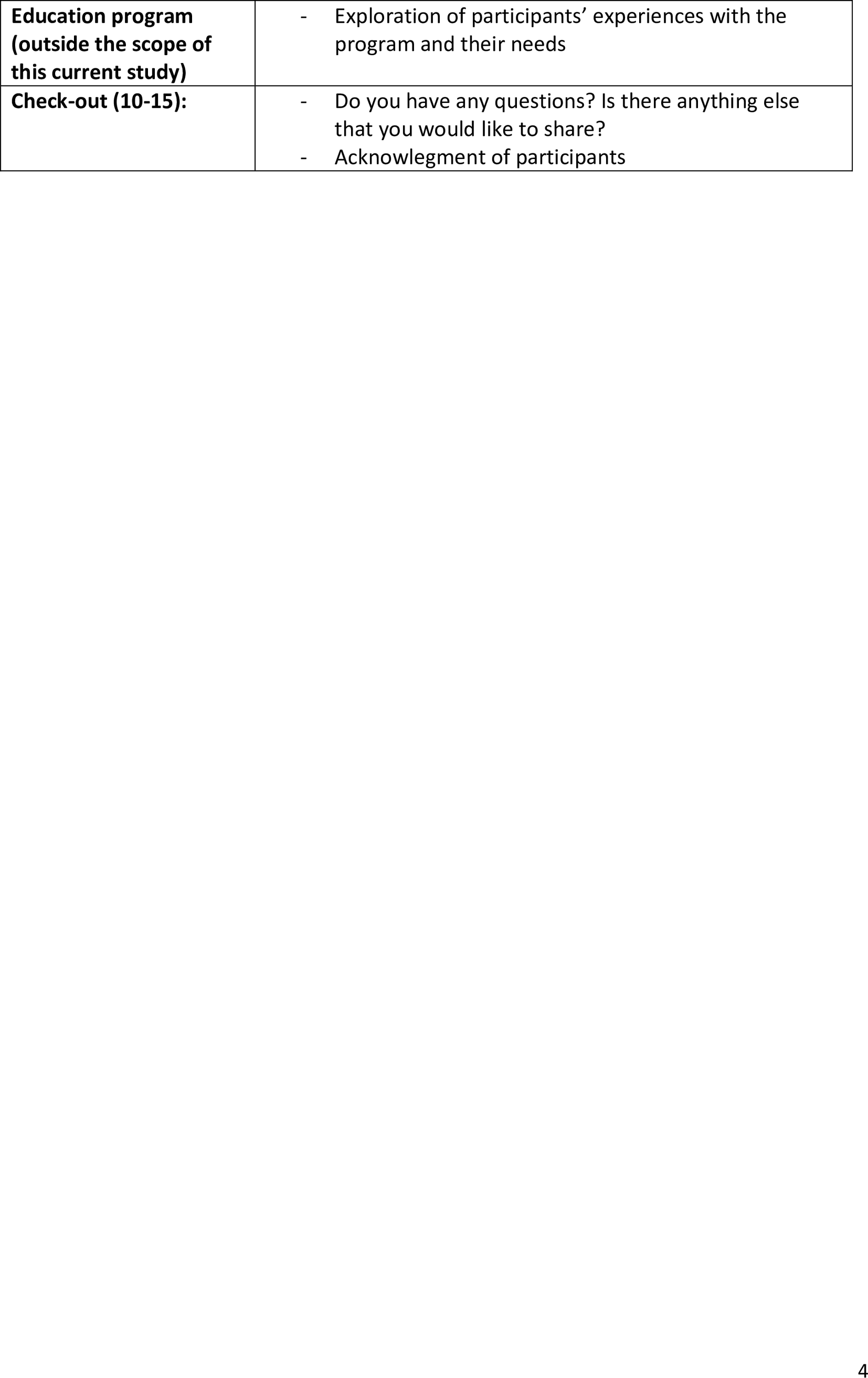

Supplement: S1 Table — (ZIP) [file pone.0315525.s001.zip › S1_Table (4).tif]
